# Pathology and Therapeutics of Cholera

**Published:** 1849-08

**Authors:** 


*Pathology and Therapeutics of Cholera.*—" There are certain points," says Dr. Garrod, in his interesting paper on the Pathological Condition of the Blood in Cholera, " with regard to the pathology and therapeutics of the disease, which the consideration of the results of the chemical examination of the blood and other fluids naturally suggests to the mind. In the first place, it would appear that the cholera poison, when introduced into the blood in sufficient quantities, causes an intense exosmotic action of the mucous membrane of the alimentary canal, at the same time destroying its endosmotic power. The blood, therefore, being deprived of a certain amount of water and salts, by the intestinal evacuations, and not possessing the power of regaining these by absorption from the stomach, becomes altered in the manner we have seen, and ill suited for circulation in the extreme vessels; thereby giving rise to suppression of the various excreting functions, by which in turn it is rendered impure. But a question now arises: Is this condition of blood essential, and cannot the stage of collapse be induced by the direct influence of the poison? There are certain cases known by the name of 'Cholera Sicca,' which would seem to favor this latter view; but from what I can ascertain, no analyses of blood have been made in such, and as far as my own experience goes, the amount of intestinal evacuations in any case is by no means an indication of the extent to which the blood has become altered. This is also well shown by the condition of the blood in severe bilious diarrhœa, in which its specific gravity appears to remain unaltered, the endosmotic or absorbing power probably remaining entire. Supposing this latter property entirely suspended, it would require but little amount of intestinal evacuation to cause this condition of blood; the loss of water by the skin and lungs would alone soon produce it; and that this power is sometimes lost, will be seen in examining Case V. (Worts,) in which, although many gallons of water were taken into the stomach, the blood still continued to increase in specific gravity.

"Assuming that such a condition of intestinal mucous membrane exists in cholera, it gives us but little hopes of effecting much by remedies administered by the mouth, during the collapse: and experience has shown us, that very little confidence can be placed in them. The saline drinks, recommended by Dr. Stevens, must here fail, as even water is unable to be absorbed. This led to the method of injection of saline fluids into the veins; and certainly it appears that, even in the most intense stage of collapse, patients may, for a time, be restored by their employment. Unfortunately, however, the improvement has, in most cases, proved but temporary; but still enough has been seen to cause many to think that their use is strongly called for. Should they be ever again employed, I think that more attention should be paid both to the nature and quantity of the salts contained in the fluid than has hitherto been done; and a solution should be employed whose composition resembles, as much as possible, the portion of the blood which has been lost. One would be apt to think that the blood could not bear with impunity a considerable quantity of carbonate of soda in place of the phosphate; yet such a substitution, I believe, has generally been made. May not the use of improper fluids have been in part the cause of the truth of the remark quoted by Dr. Watson, in his *Lectures on the Practice of Medicine*, that, 'However it might be with pigs and herrings, *salting* a patient in cholera was not always the same thing as *curing* him.'

"Might not some agent be injected, which would tend to prevent the exosmotic action of the intestines? Certain bodies, possessing such a power on membranes, have been found. When reaction takes place, and the watery portion of the blood becomes restored, it would then seem rational to employ drinks containing small quantities of the salts; for it does not seem improbable that the saline deficiency, which must then occur, unless supplied, may tend to prevent the due action of the kidneys and other excreting organs. At this time, also, other remedies, as calomel, etc., should be given, with the intention of restoring the excretions."—*London. Journ. Med.*

